# Socioeconomic differences in waiting times for elective surgery: a population-based retrospective study

**DOI:** 10.1186/1472-6963-12-268

**Published:** 2012-08-21

**Authors:** Alessio Petrelli, Giuliana De Luca, Tania Landriscina, Giuseppe Costa

**Affiliations:** 1 Epidemiology and Public Health Unit, Piedmont Region, Turin, Italy; 2 Department of Economics and Statistics, University of Calabria, Cosenza, Italy; 3 Department of Hygiene and Public Health, University of Turin, Turin, Italy

**Keywords:** Equity, Access, Duration analysis, Waiting times, Socioeconomic status

## Abstract

**Background:**

Widespread literature on inequity in healthcare access and utilization has been published, but research on socioeconomic differences in waiting times is sparse and the evidence is fragmentary and controversial. The objective of the present study is the analysis of the relationship between individual socioeconomic level and waiting times for in-hospital elective surgery.

**Methods:**

We retrospectively studied the waiting times experienced by patients registered on hospital waiting lists for 6 important surgical procedures by using the Hospital Discharge Database (HDD) of the Piedmont Region (4,000,000 inhabitants in the North West of Italy) from 2006 to 2008. The surgical procedures analyzed were: coronary artery by-pass (CABG), angioplasty, coronarography, endarterectomy, hip replacement and cholecystectomy. Cox regression models were estimated to study the relationship between waiting times and educational level taking into account the confounding effect of the following factors: sex, age, comorbidity, registration period, and Local Health Authorities (LHA) as a proxy of supply.

**Results:**

Median waiting times for low educational level were higher than for high educational level for all the selected procedures. Differences were particularly high for endarterectomy and hip replacement. For all considered procedures, except CABG, an inverse gradient between waiting times and educational level was observed: the conditional probabilities of undergoing surgery were lower among individuals with a low to middle level education than for individuals with a higher level of education after adjustment for sex, age, comorbidities, registration period, and LHAs. For most procedures the effect decreases over the follow up period.

**Conclusions:**

The results of the study show evidence of inequalities in access to elective surgery in Italy. Implementation of policies aimed to promote national information initiatives that guarantee wider access to those with low socio-economic status is strongly recommended.

## Background

Over recent decades, long waiting lists for elective surgery have become an issue of great relevance for several National Health Services [1]. Waiting lists are used as a mechanism to allocate scarce resources, but health care systems should aim to provide healthcare to those with the greatest need first, in order to prevent adverse outcomes [2]. To this purpose, in recent years the health authorities of several western countries, United Kingdom, New Zealand, Canada, Sweden, Norway and Italy amongst others, have promoted several initiatives mainly aimed at supply but, as in the case of New Zealand, Canada and Italy these initiatives include strategies for demand, patient prioritization on the basis of clinical conditions and maximal waiting times to be met [3,4]. The mechanism should be independent from the socioeconomic status in order to guarantee equity of access.

Barriers generated by factors relating to both supply and demand may still prevent access, thereby bringing about an exacerbation of existing inequalities in health. The former may be due to financial factors such as out-of-pocket money, insurance coverage, the existence of health benefit packages, geographical factors such as distance from health care resources, or organizational factors such as opening hours and waiting time [5]. The latter deals with individual characteristics, including age, gender, ethnicity, income, education, health literacy, communication skills and health status perception, which may affect healthcare-seeking behaviour [6]. Because of these barriers, more vulnerable groups may suffer much more than others [7]. The relative weight of the various access barriers should be disentangled in order to design effective measures for limiting access inequalities.

Unlike the widespread existing literature on inequity in healthcare access and utilization [8-12], research on socioeconomic differences in waiting times is less systematic and the evidence is fragmentary and controversial. Some studies conducted in Northern Europe and North America during the previous decade have shown an inverse socioeconomic trend, as in Scotland for cardiac surgery [13] and in England for ophthalmologic surgery and hip replacement [14,15], whilst others have found no or a weak association, as in Canada [16], Australia [17] and Norway [18]. A large retrospective English study shows decreasing inequalities in waiting times during the last decade for elective hip replacement and cataract repair [19].

Two recent studies show wider socioeconomic differentials [20,21]. The first study, based on the International Survey of Health, Ageing and Retirement in Europe (SHARE), provides evidence of inequity in waiting times that favour the more educated patients in seven European countries (Denmark, France, Greece, Italy, the Netherlands, Spain and Sweden). The inverse association between education levels and waiting times is evident regardless of the organizational characteristics of health systems. In Denmark, the Netherlands and Sweden for non-urgent inpatient/outpatient surgery, individuals with higher levels of education wait significantly less than individuals with lower levels. Income is also negatively associated with waiting times in Greece for non-emergency surgery.

The second study is based on administrative data (2001 National Hospital Episode Statistics data) and produces evidence of an inverse association between socioeconomic status and waiting times for elective total hip replacement in England using an aggregate deprivation indicator.

In Italy, research in the area of equity in waiting lists is limited to a study that found an inverse relationship between socioeconomic status and waiting time for surgery after hip fracture, using an aggregate indicator of socioeconomic status, but only using pre-surgery length of stay as a proxy measure of the waiting time [22].

The purpose of this study is to analyze the relationship between individual socioeconomic level and waiting time for in-hospital elective surgery.

## Methods

### Record selection and data collection

We retrospectively studied the waiting times experienced by patients registered on hospital waiting lists for 6 of the 14 surgical procedures identified by the “National monitoring plan to reduce waiting time for elective surgery”, by using the **Hospital Discharge Database (**HDD) of the Piedmont Region (4,463,000 inhabitants in the North West of Italy) from 2006 to 2008. The capital of Piedmont is Turin (907,000 inhabitants).

In Italy the health service is universalistic: each citizen has the right to health care, free of charge or following a co-payment fee, for a wide range of health problems [23] Enrolment on the elective surgery waiting list occurs directly, through the specialist hospital physician or through the patient contacting the chosen hospital under the guidance of the general practitioner. The National Health System is organized on a regional basis: the Regional Health System (RHS) of Piedmont Region is divided into Local Health Authorities (LHA), which provide medium and low-complexity hospital-based healthcare and community care, and Hospital Trusts (HT), providing high-complexity hospital care. Finally, there are a small number of private hospitals, mainly religious, funded by the RHS, that provide medium or low-complexity care.

The HDD is the administrative database that covers all episodes of care for hospital patients (n = 820,000) provided in the hospitals funded by the RHS. The HDD includes information on admission and discharge dates, coexisting medical conditions, patients’ characteristics (sex and age), diagnosis codes (up to 5 digits), surgical procedure codes (up to 5 digits) according to the International Classification of Disease, Ninth Revision, Clinical Modification (ICD-9-CM), the patient’s LHA of hospitalisation and educational level, classified into seven categories. The database is directly managed by the regional authority for information system. The study population consisted of patients undergoing surgery between 2006 and 2008. It is worth noting that since the HDD records discharges some individuals could be registered before 2006.

The surgical procedures analyzed were: coronary artery by-pass (CABG), angioplasty, coronarography, endarterectomy, hip replacement and cholecystectomy. The time on the waiting list was computed as the number of days from registration (the decision to treat or referral) to surgery.

Exclusion criteria for the present study were: 1) missing values from the education variable; 2) providers with very few cases; 3) waiting times higher than the 99^th^ percentile.

The percentage of missing values from the education variable for the overall sample including all hospitalisations for the 6 selected procedures was 34.6%, 51.3%, 45.9%, 51.3%, 34.2%, 26.2% respectively for CABG, angioplasty, coronarography, endarterectomy, hip replacement and cholecystectomy. In order to reduce this percentage we conducted two record linkage activities: 1) by means of anonymous record linkage between the HDDs over the period under consideration (2006–2008), we were able to reduce the percentage of missing values by attributing the highest level of education reported for the three years of observation, 2) exclusively for residents in Turin, a record linkage based on gender, place and date of birth was conducted between our dataset and the municipal registry, which included information about educational level. After the linkage process, missing values dropped to 12.6% for CABG, 24.6% for angioplasty, 21.8% for coronarography, 24.2% for endarterectomy, 16.1% for hip replacement and 17.4% for cholecystectomy. Table 1 summarises the cases recovered by types of record linkage and the cases excluded from the analysis by reasons for exclusion for each operation.

**Table 1:** Sample selection criteria and resulting sample size

**Surgery**	**Initial sample**	**Records with missing values for the education variable**	**Missing values recovery from record linkage between the HDDs**	**Missing values recovery from record linkage with the Turin municipal registry**	**Providers with few cases**	**Waiting times higher than the 99**^**th**^**percentile**	**Final sample**
**CABG**	4,465	1,543	926	56	2	40	3,862
**Angioplasty**	9,973	5,115	1,770	893	16	77	7,428
**Coronarography**	26,307	12,081	4,693	1,640	36	213	20,310
**Endarterectomy**	4,126	2,115	731	386	8	33	3,087
**Hip replacement**	15,982	5,462	2,245	639	10	134	13,260
**Cholecystectomy**	17,246	4,511	732	783	24	143	14,083

### Statistical analysis

In order to make the results comparable with international classification, educational level was grouped into three categories: no education to primary education; lower secondary education; upper secondary education and higher education (reference category).

To examine the relation between waiting times and education, we first estimated the cumulative probability of undergoing surgery by waiting time since registration using the product-limit method. The log-rank test was used to compare waiting times across education groups. Cox proportional hazard models [24] without censored data were then estimated to study the relationship between waiting times and educational level taking into account the confounding effect of sex (reference: female), age (reference: > = 75), and registration period (reference: 2006) [25,26]. Moreover, in order to take into account differences in the severity of clinical conditions, models were adjusted for the Charlson Comorbidities Index (CCI) [27,28] (reference = no comorbidities).

Lastly, LHAs (reference: LHA - city of Turin) were added in the models to adjust for possible variations in geographical and access management. We estimated models by progressively adding subsets of covariates according to the following pattern: 1) education; 2) age; 3) CCI; 4) LHAs and 5) registration period. Table 2 describes the subset of variables used in the analysis along with their codes and values.

**Table 2:** Data dictionary for the variables used in the study

**Description**	**Codes/Values**
Waiting time from registration to surgery	Integer for number of days
Education (at surgery time)	Primary school or less (LOW)
	Middle school (MIDDLE)
	Upper secondary school or higher education (HIGH)
Gender	Male
	Female
Age groups (at surgery)	<= 54
	55-64
	65-74
	> = 75
Year of registration	<2006
	2006
	2007
	2008
CCI	0,1,or 2 (≥2)
LHA/HT	LHA- City of Turin; LHA - Western Piedmont; LHA - Northern Piedmont; LHA - Southern Piedmont; LHA - Eastern Piedmont; HT - City of Turin; HT - Piedmont Region; Other Hospitals.

The models were compared using the Akaike information criteria. The assumption of proportional hazards was evaluated:

1) adding to the model the interaction of the covariate with the time for each variable and testing the statistical significance with the Wald test;

2) through the Schoenfeld residual graphic obtained from the model without the interaction term.

The instantaneous probability of undergoing surgery at time *t*, given that a patient has not been operated yet, was expressed as:

(1) $$h\left(t,x\right)=h_{0}\left(t\right)\exp\left(\sum_{j}\beta_{j}x_{j}\right)j=1,2,\ldots ,n$$

where $h_{0}\left(t\right)$ is the hazard rate at time *t* for a reference subject and *β*_*j*_ is the log hazard ratio associated with covariate *j*. To simplify, assume that there is one covariate *x*, then the hazard ratio between two subjects with covariate values *x*_1_ and *x*_2_ is:

(2) $$HR=\frac{h_{x_{2}}\left(t\right)}{h_{x_{1}}\left(t\right)}=\exp\left[\beta \left(x_{2}-x_{1}\right)\right]\text{.}$$

When $x_{2}=x_{1}+1$ the hazard ratio is equal to $HR=\exp\left(\beta_{j}\right)$ and measures the effect of one unit increase in *x* on the probability of undergoing surgery. If the HR is greater than 1 (*β*_*j*_*>*0), the probability of undergoing surgery increases for subjects with covariate value *x*_2_ compared to subjects with covariate value *x*_1_, while a HR lower than 1 (*β*_*j*_*<*0) indicates a decreased probability of leaving the waiting list. The HR is assumed to be constant over time. To assess this assumption we add a time-varying coefficient *γ* in the hazard function:

(3) $$h_{0}\left(t\right)=h_{0}\left(t\right)\exp\left(\beta x+\gamma xg\left(t\right)\right)$$

The hazard ratio for a unit increase in the variable *x* is:

(4) $$HR\left(t\right)=\frac{h_{x+1}\left(t\right)}{h_{x}\left(t\right)}=\exp\left[\left(\beta +\gamma xg\left(t\right)\right)\right]$$

where *γ* measures the change in the hazard ratio with time, i.e. non-proportionality. If *γ>0* (*γ<0*) than the HR increases (decreases) over time. Testing for non-proportionality of the hazards is equivalent to testing whether *γ* is significantly different from zero. No ethical approval was required by Italian law 211/2003 which explains why no ethic committee’s permission is needed for this kind of study in Italy (anonymous data from administrative database).

## Results

### Characteristics of the population

As shown in Table 3, during the study period, most patients who underwent hip replacement (54.7%) and cardiovascular procedures (CABG, 49.1%; angioplasty, 43.2%; coronarography, 46.2%; endarterectomy, 63.1%) were in the group with a lower educational level. Cholecystectomy was balanced across education groups. Most subjects undergoing cardiovascular procedures were male whereas most of those undergoing cholecystectomy and hip replacement were female. At registration, the majority of patients, among those who underwent cardiovascular procedures, were aged 65 and over. The reverse was observed for cholecystectomy, where younger patients underwent more surgery than older ones. The proportion of patients with at least a comorbidity at the time of the registration in the list were particularly high, as expected, among subjects awaiting cardiovascular procedures and, to a lesser extent, among those performing hip replacement and cholecystectomy. As for the geographical location, it is worth noticing that 34.2% of CABGs were performed at the HT of the Piedmont Region; 31.7% of angioplasties and 23.4% of coronarographies at Turin LHA; about two thirds of endarterectomy procedures at the two regional Health Trusts, around 20% of hip replacement surgery at the LHA of Southern Piedmont; and 21.7% of cholecystectomies at Northern Piedmont LHA.

**Table 3:** Characteristics of cohorts

	**CABG**		**Angioplasty**		**Coronarography**		**Endoarterectomy**		**Hip replacement**		**Cholecystectomy**	
	**No.**	**%**	**No.**	**%**	**No.**	**%**	**No.**	**%**	**No.**	**%**	**No.**	**%**
**Education**												
Low education	1896	49.1	3207	43.2	9375	46.2	1947	63.1	7257	54.7	4757	33.8
Middle education	1124	29.1	2277	30.7	5987	29.5	692	22.4	3882	29.3	5366	38.1
High education	842	21.8	1944	26.2	4948	24.4	448	14.5	2121	16.0	3960	28.1
**Gender**												
Male	2908	75.3	5719	77.0	13983	68.8	2115	68.5	5580	42.1	5407	38.4
Female	954	24.7	1709	23.0	6327	31.2	972	31.5	7680	57.9	8676	61.6
**Age groups**												
<55	329	8.5	791	10.6	2419	11.9	67	2.2	1391	10.5	6041	42.9
55-64	861	22.3	1897	25.5	4965	24.4	404	13.1	2450	18.5	3140	22.3
65-74	1610	41.7	2949	39.7	7980	39.3	1290	41.8	5162	38.9	3368	23.9
>74	1062	27.5	1791	24.1	4946	24.4	1326	43.0	4257	32.1	1534	10.9
**CCI**												
0	2712	70.2	4494	60.5	14136	69.6	2165	70.1	12477	94.1	12561	89.2
1	761	19.7	2253	30.3	4811	23.7	645	20.9	629	4.7	946	6.7
2+	389	10.1	681	9.2	1363	6.7	277	9.0	154	1.2	576	4.1
**Year of registration**												
<2006	72	1.9	102	1.4	291	1.4	115	3.7	1567	11.8	610	4.3
2006	1267	32.8	2368	31.9	6583	32.4	1046	33.9	4342	32.7	4656	33.1
2007	1304	33.8	2432	32.7	6740	33.2	983	31.8	4273	32.2	4803	34.1
2008	1219	31.6	2526	34.0	6696	33.0	943	30.5	3078	23.2	4014	28.5
**Location**												
LHA - City of Turin	261	6.8	2355	31.7	4747	23.4	375	12.1	2129	16.1	1971	14.0
LHA - Province of Turin			511	6.9	900	4.4	119	3.9	1306	9.8	1961	13.9
LHA - Eastern Piedmont	615	15.9	734	9.9	3102	15.3	105	3.4	1258	9.5	1230	8.7
LHA - Southern Piedmont			158	2.1	581	2.9	57	1.8	2584	19.5	1594	11.3
LHA - Northern Piedmont	922	23.9	1049	14.1	3842	18.9	395	12.8	2151	16.2	3060	21.7
HT – City of Turin	742	19.2	1509	20.3	3275	16.1	1055	34.2	1651	12.5	2477	17.6
HT - Piedmont Region	1322	34.2	1112	15.0	3863	19.0	981	31.8	770	5.8	921	6.5
Other Hospitals									1411	10.6	869	6.2
**Total**	3862	100.0	7428	100.0	20310	100.0	3087	100.0	13260	100.0	14083	100.0

*CABG* = Coronary Artery Bypass Graft, *CCI* = Charlson Comorbidity Index, *LHA* = Local Health Authority, *HT* = Health Trust.

Table 4 summarizes median waiting times of the cohort for the selected elective surgeries by education. Median waiting times for lower educational levels were higher than for higher educational levels for all the selected procedures. Differences were particularly high for endarterectomy and hip replacement. Mean waiting times were systematically greater than median waiting times, indicating that the distribution was highly skewed (data not shown). High geographical heterogeneity was also observed between LHAs.

**Table 4:** Median waiting times and 95% CI for variables used in the study

	**CABG**	**Angioplasty**	**Coronarography**	**Endarterectomy**	**Hip replacement**	**Cholecystectomy**
**Education**						
None/low Education	12.0 [11.5,12.5]	8.0 [7.6,8.4]	8.0 [7.8,8.2]	28.0 [26.3,29.7]	83.0 [80.5,85.5]	32.0 [30.9,33.1]
Middle Education	12.0 [11.4,12.6]	7.0 [6.5,7.5]	7.0 [6.7,7.3]	27.0 [24.1,29.9]	76.0 [72.8,79.2]	32.0 [31.0,33.0]
High Education	10.0 [9.5,10.5]	6.0 [5.7,6.3]	7.0 [6.8,7.2]	20.0 [17.3,22.7]	60.0 [55.6,64.4]	31.0 [29.6,32.4]
**Gender**						
Male	11.0 [[10.7,11.4]	7.0 [6.7,7.3]	7.0 [6.8,7.2]	26.0 [24.5,27.5]	78.0 [75.2,80.8]	31.0 [30.0,32.0]
Female	11.0 [10.4,11.6]	7.0 [6.6,7.4]	7.0 [6.8,7.2]	28.0 [25.7,30.3]	77.0 [74.7,79.3]	33.0 [32.1,33.9]
**Age groups**						
<= 54	11.0 [10.0, 12.0]	7.0 [6.3,7.7]	8.0 [7.6,8.4]	22.0 [16.6,28.4]	74.0 [68.5,79.5]	33.0 [32.0,34.0]
55-64	11.0 [10.4,11.6]	7.0 [6.5,7.5]	7.0 [6.7,7.2]	24.0 [20.6,27.4]	83.0 [78.4,87.6]	33.0 [31.5,34.5]
65-74	12.0 [11.5,12.5]	7.0 [6.6,7.4]	7.0 [6.8,7.2]	26.0 [23.8,28.2]	84.0 [81.0,87.0]	32.0 [30.6,33.4]
> = 75	11.0 [10.4,11.6]	7.0 [6.6,7.4]	7.0 [6.8,7.2]	28.0 [26.1,29.9]	69.0 [66.1,71.9]	27.0 [25.4,28.6]
**CCI**						
0	11.0 [10.7,11.3]	7.0 [6.7,7.3]	7.0 [6.9,7.1]	24.0 [22.6,25.4]	76.0 [74.1,77.9]	32.0 [31.3,32.7]
1	12.0 [11.1,13.0]	7.0 [6.4,7.6]	7.0 [6.6,7.4]	31.0 [27.5,34.5]	100.0 [92.1,107.9]	36.0 [33.2,38.8]
> = 2	12.0 [10.6,13.4]	10.0 [8.6,11.4]	10.0 [8.9,11.0]	36.0 [29.4,42.5]	91.5 [76.0,107.0]	22.0 [20.2,23.8]
**Year of registration**						
<2006	18.0 [14.4,,21.6]	34.0 [24.9,43.1]	36.0 [31.1,40.9]	85.0 [74.5,95.5]	194.0 [185.4,202.6]	76.0 [69.9,82.1]
2006	11.0 [10.6,11.4]	7.0 [6.6,7.4]	7.0 [6.8,7.2]	25.0 [22.7,27.3]	83.0 [79.9,86.1]	32.0 [31.0,33.0]
2007	12.0 [11.4,12.6]	7.0 [6.6,7.4]	7.0 [6.7,7.3]	27.0 [24.6,29.4]	77.0 [74.1,80.0]	32.0 [30.7,33.3]
2008	11.0 [10.5,11.5]	7.0 [6.6,7.4]	7.0 [6.8,7.2]	24.0 [22.1,25.9]	41.0 [39.0,43.0]	28.0 [27.0,29.0]
**LHA**						
LHA – City of Turin	7.0 [6.5,7.5]	6.0 [5.9,6.1]	5.0 [4.9,5.1]	22.0 [16.5,27.5]	50.0 [45.8,54.2]	36.0 [33.4,38.6]
LHA – Province of Turin		29.0 [27.2,30.8]	28.0 [26.6,29.4]	20.0 [18.8,21.2]	79.5 [75.3,83.7]	35.0 [33.3,36.7]
LHA – Eastern Piedmont	12.0 [11.2,12.8]	10.0 [8.9,11.1]	8.0 [7.6,8.4]	11.0 [9.7,12.3]	27.5 [24.6,30.3]	24.0 [22.3,25.7]
LHA – Southern Piedmont	-	8.0 [6.9,9.1]	9.0 [8.1,9.9]	32.0 [25.5,38.5]	106.0 [102.2,109.8]	30.0 [28.7,31.3]
LHA – Northern Piedmont	11.0 [10.5,11.5]	10.0 [9.4,10.6]	10.0 [9.8,10.2]	16.0 [14.6,17.4[	29.0 [26.7,31.3]	25.5 [24.8,26.2]
HT – City of Turin	11.0 [9.7,12.3]	8.0 [7.3,8.7]	13.0 [12.3,13.7]	34.0 [29.9,38.1]	121.0 [114.5,127.5]	38.0 [35.6,40.4]
HT – Piedmont Region	13.0 [12.4,13.6]	4.0 [3.8,4.2]	4.0 [3.8,4.2]	33.0 [30.7,35.3]	132.5 [123.5,141.5]	52.0 [43.4,60.6]
Other Hospitals	-	-	-	-	118.0 [112.4,123.6]	53.0 [48.6,57.4]
**Total**	11.0 [10.7,11.3]	7.0 [6.8,7.2]	7.0 [6.9,7.1]	26.0 [24.7,27.3]	77.0 [75.2,78.8]	32.0 [31.3,32.7]

*CI* = confidence interval, *CABG* = Coronary Artery Bypass Graft, *CCI* = Charlson Comorbidity Index, *LHA* = Local Health Authority, *HT* = Health Trust.

### Statistical models

As measured by the log-rank test, statistically significant differences (p < 0.001) in access to surgery between education groups were observed for CABG, angioplasty, coronarography, endarterectomy and hip replacement, with shorter waiting times for individuals with a higher education level (data not shown).

Table 5 presents the results of fitting for the main effects and Table 6 shows linear-time interaction coefficients with educational level.^1^ For all considered procedures except for CABG an inverse and statistically significant gradient between waiting times and educational level was observed: the conditional probabilities of undergoing surgery were lower among people with low and middle education levels than for more highly educated people after adjustment for sex, age, comorbidities, registration period, and LHAs.

**Table 5:** Hazard ratios and 95% CI for Cox proportional hazards models

	**CABG**	**Angioplasty**	**Coronarography**	**Endarterectomy**	**Hip replacement**	**Cholecystectomy**
**Education**						
Low education	0.940 [0.860 - 1.026]	0.880 [0.819 - 0.944]	0.920 [0.883 - 0.958]	0.660 [0.571 - 0.762]	0.761 [0.709 - 0.817]	0.838 [0.788 - 0.892]
Middle education	0.927 [0.846 - 1.015]	0.905 [0.841 - 0.974]	0.956 [0.920 - 0.993]	0.745 [0.631 - 0.880]	0.852 [0.791 - 0.918]	0.925 [0.875 - 0.978]
**Gender**						
Male	1.008 [0.935 - 1.088]	0.929 [0.879 - 0.982]	1.004 [0.974 - 1.035]	1.086 [1.005 - 1.174]	0.980 [0.946 - 1.015]	1.043 [1.008 - 1.080]
**Age groups**						
<55	1.148 [1.009 - 1.307]	0.979 [0.898 - 1.068]	0.926 [0.880 - 0.974]	1.065 [0.825 - 1.374]	0.927 [0.869 - 0.989]	0.824 [0.774 - 0.877]
55-64	1.100 [1.002 - 1.209]	0.962 [0.900 - 1.028]	0.960 [0.921 - 0.999]	1.046 [0.934 - 1.172]	0.847 [0.804 - 0.891]	0.833 [0.782 - 0.887]
65-74	1.019 [0.942 - 1.102]	0.956 [0.901 - 1.014]	0.976 [0.942 - 1.012]	1.015 [0.939 - 1.097]	0.839 [0.795 - 0.885]	0.856 [0.805 - 0.909]
**CCI**						
1	0.989 [0.908 - 1.078]	0.881 [0.837 - 0.929]	0.886 [0.857 - 0.917]	0.787 [0.692 - 0.894]	0.917 [0.846 - 0.995]	0.876 [0.799 - 0.962]
> = 2	1.004 [0.895 - 1.127]	0.681 [0.614 - 0.755]	0.693 [0.646 - 0.743]	0.855 [0.751 - 0.973]	0.763 [0.592 - 0.984]	1.785 [1.596 - 1.997]
**Year of registration**						
<2006	0.713 [0.562 - 0.906]	0.348 [0.265 - 0.458]	0.406 [0.361 - 0.457]	0.229 [0.162 - 0.324]	0.272 [0.245 - 0.301]	0.406 [0.358 - 0.459]
2007	0.876 [0.810 - 0.947]	1.025 [0.968 - 1.085]	0.992 [0.958 - 1.026]	0.967 [0.855 - 1.095]	1.118 [1.050 - 1.189]	0.929 [0.892 - 0.967]
2008	0.915 [0.845 - 0.991]	0.946 [0.886 - 1.011]	1.018 [0.984 - 1.054]	1.022 [0.903 - 1.158]	1.779 [1.659 - 1.908]	1.114 [1.068 - 1.163]
**Location**						
LHA - Province of Turin		0.235 [0.206 - 0.267]	0.237 [0.216 - 0.260]	0.457 [0.319 - 0.655]	0.607 [0.554 - 0.666]	1.161 [1.072 - 1.257]
LHA - Eastern Piedmont	0.326 [0.268 - 0.398]	0.540 [0.495 - 0.589]	0.613 [0.585 - 0.643]	1.867 [1.500 - 2.323]	1.334 [1.243 - 1.432]	1.491 [1.388 - 1.603]
LHA - Southern Piedmont		0.705 [0.599 - 0.830]	0.490 [0.437 - 0.548]	0.964 [0.729 - 1.276]	0.369 [0.340 - 0.400]	1.329 [1.221 - 1.448]
LHA - Northern Piedmont	0.467 [0.396 - 0.551]	0.518 [0.480 - 0.559]	0.533 [0.510 - 0.558]	1.411 [1.222 - 1.629	1.248 [1.175 - 1.326]	1.621 [1.511 - 1.738]
HT - City of Turin	0.472 [0.395 - 0.565]	0.580 [0.543 - 0.620]	0.383 [0.363 - 0.405]	0.594 [0.526 - 0.671]	0.355 [0.323 - 0.389]	0.917 [0.864 - 0.973]
HT - Piedmont Region	0.478 [0.414 - 0.552]	1.600 [1.455 - 1.759]	1.181 [1.120 - 1.245]	0.617 [0.535 - 0.711]	0.392 [0.348 - 0.443]	0.728 [0.652 - 0.812]
Other Hospitals					0.318 [0.288 - 0.352]	0.624 [0.558 - 0.698]

*CI* = confidence interval, *CABG* = Coronary Artery Bypass Graft, *CCI* = Charlson Comorbidity Index, *LHA* = Local Health Authority, *HT* = Health Trust.

**Table 6:** Hazard ratios and 95% CI for interaction between time and covariates (Cox proportional hazards models)*

	**CABG**	**Angioplasty**	**Coronarography**	**Endarterectomy**	**Hip replacement**	**Cholecystectomy**
**Education**						
Low education		1.004 [1.001 - 1.006]	1.001 [1.000 - 1.003]	1.005 [1.002 - 1.007]	1.001 [1.001 - 1.002]	1.001 [1.000 - 1.002]
Middle education		1.002 [1.000 - 1.005]		1.004 [1.001 - 1.007]	1.001 [1.000 - 1.001]	1.001 [1.000 - 1.002]
**Age groups (years)**						
65-74					1.001 [1.000 - 1.001]	
**CCI**						
1				1.002 [1.000 - 1.004]		1.001 [1.000 - 1.002]
> = 2		1.004 [1.001 - 1.007]	1.004 [1.002 - 1.006]		1.002 [1.000 - 1.004]	0.997 [0.995 - 0.999]
**Year of registration**						
<2006		1.004 [1.000 - 1.009]		1.012 [1.009 - 1.016]	1.003 [1.002 - 1.003]	1.001 [1.001 - 1.002]
2007				1.003 [1.001 - 1.005]	1.000 [0.999 - 1.000]	
2008		1.004 [1.001 - 1.006]		1.004 [1.001 - 1.006]		
**Location**						
LHA - Province of Turin		1.012 [1.009 - 1.015]	1.010 [1.008 - 1.012]	1.060 [1.046 - 1.075]	1.004 [1.003 - 1.004]	1.001 [1.000 - 1.002]
LHA - Eastern Piedmont	1.030 [1.021 - 1.040]					
LHA - Southern Piedmont			1.007 [1.003 - 1.011]		1.005 [1.005 - 1.006]	1.005 [1.004 - 1.006]
LHA - Northern Piedmont	1.022 [1.015 - 1.030]					1.001 [1.000 - 1.002)
HT - City of Turin	0.992 [0.986 - 0.997]		1.006 [1.005 - 1.008]		1.004 [1.003 - 1.004]	
HT - Piedmont Region		0.972 [0.964 - 0.979]	0.979 [0.976 - 0.982]	1.003 [1.001 - 1.004]	1.004 [1.004 - 1.005]	1.003 [1.002 - 1.004]
Other Hospitals					1.006 [1.005 - 1.006]	1.004 [1.003 - 1.005]

*CI* = confidence interval, *CABG* = Coronary Artery Bypass Graft, *CCI* = Charlson Comorbidity Index, *LHA* = Local Health Authority, *HT* = Health Trust.

*: only statistically significant effects.

The interaction terms between education and follow up time are statistically significant for all procedures except for CABG suggesting that the adjusted hazard ratios associated with education were not constant over time (Table 6). Specifically, hazard ratios higher than 1 show that the difference of probability of undergoing surgery between the two groups decreases with increasing follow up.

In models with statistically significant interactions, both the low and middle level education groups at the start of follow-up show a lower probability of leaving the waiting list compared to the higher level education group with the hazard ratio for the low level education group being slightly lower at the start of follow up compared to the middle level group, statistically significant for all procedures except for CABG. At the start of follow up (Table 5), subjects with a middle level of education registered for angioplasty, coronarography, endarterectomy, hip replacement, cholecystectomy have access to surgery at a rate that is respectively 9.5%, 4.4%, 25.5%, 14.8%, 7.5% lower than for subjects with a higher level of education. The estimated gap between the two groups at the median waiting time decreases to 8.2% for angioplasty, 17.4% for endarterectomy, 8% for hip replacement and 4.5% for cholecystectomy, while for coronarography the estimated gap is constant over time.

Similarly, subjects with a lower education level registered for angioplasty, coronarography, endarterectomy, hip replacement and cholecystectomy have access to surgery at a rate that is respectively 12.0%, 8%, 34%, 23.9%, 16.2% lower than for subjects with a higher education. The estimated difference between lower and higher education in terms of median waiting time decreases to 9.5% for angioplasty, 7.4% for coronarography, 24.9% for endarterectomy, 17.8% for hip replacement, 13.5% for cholecystectomy. The reduction is more pronounced in the lower level education group compared to the middle level education group for angioplasty and endarterectomy but is less pronounced for hip replacement and cholecystectomy. Moreover, the chance of delayed surgery increases for males undergoing angioplasty while it decreases for males awaiting endarterectomy and cholecystectomy. The probability of longer waiting times for surgery are statistically significant for older patients scheduled for CABG while they are shorter for older patients waiting for coronarography, hip replacement and cholecystectomy. Coexisting illnesses extend waiting times for most procedures as well.

The effect of the registration period is also statistically significant for most procedures and tends to decrease over time. More precisely, patients registered after 2006 for hip replacement and cholecystectomy (2008 only) have a higher probability of waiting less than those on the list in 2006 whereas the opposite was observed for those registered for CABG after 2006.

Lastly, the results show a large geographical variation in elective surgery rates for all surgical procedures with differences not spatially structured and often changing over time. Such differences may reflect variations in clinical judgment or resource levels.

### Sensitivity analysis

In order to study the risk of bias due to missing values we carried out a sensitivity analysis comparing the distribution of the covariates and the outcome in the subgroups made up respectively of subjects with:

The ascertained education level;

The education level attributed through record linkage;

The missing education level not attributed through record linkage.

The distributions result as being highly homogeneous between them and, in any case, no statistically significant differences were observed; so it is unlikely that the presence of missing values may produce relevant bias in the results.

Similarly, to exclude the risk of bias due to record linkage through the City of Turin municipal registry, we observed that the distribution of the educational level of hospitalised individuals obtained using record linkage is similar to that of hospitalised Turin residents with valid values. **The results are not** shown but are available on request.

## Discussion

To our knowledge, this study is the first to examine the relation between education and waiting times, using a population-based database and individual measure for socioeconomic status. Piedmont is the only Italian Region for which it is possible to use an individual indicator of educational level in so large a population. There are several important findings in this study. First, a lower education level is significantly associated with longer waiting times. This association persists after adjustment for demographic variables, comorbidity, registration period and supply. The effect of education on the waiting time changes with time: a lower education level has the largest effect, however over time this effect decreases.

Several issues can be cited to explain the results: differences in composition of social networks can determine a heterogeneous level of access to information. Individuals with a lower education level could face higher transaction costs (information and search costs) when choosing among alternative providers. Moreover, more educated people looking for alternative providers may have access to direct information about the waiting times for single hospitals, for example through relationships with health professionals. They may be more able to negotiate with them, to express themselves, explain their health problems and make care choices. By contrast, individuals with a lower education level are less able to overcome bureaucratic hurdles and navigate complex modern healthcare systems, to deal with several aspects of healthcare administration ranging from registration to form submission. Also, individuals with a lower education level may be less able to keep in touch with the hospital and make or attend appointments for admission. They could also experience greater difficulty in having their rights guaranteed due to lack of information on their basic rights and ways in which to access healthcare [21].

Such differences may reflect variations in clinical judgment or resource levels.

Furthermore, since socioeconomic inequities reduce over time, the longer the waiting time, the more these mechanisms seem to act in a more significant way. While the measurement of equal access to health care is a complex task involving multidimensional aspects of both access and equity [29], these results show evidence of inequalities in access to elective surgery in Italy.

### Implications and recommendations

The study involves the implementation of policies aimed at tackling inequity, acting through revision of enrolment mechanisms in waiting lists in order to reduce inequity in access [30]. In particular, a policy implication for the government is to simplify access to comparative information on waiting times across different providers. Indeed, in Italy a booking system is being implemented: general practitioners will be able to access on-line information of waiting times across providers and book patients directly with the provider with the shortest waiting times. This might reduce inequalities in waiting times by reducing the differences for accessing information systems.

The results of the study also suggest the need for further investigations aimed at exploring the association between waiting times and outcome of care; in fact, if waiting times are a likely determinant of worse quality of care, they may well assume the role of determinants that could amplify the socioeconomic differences in the outcome of medical treatments.

### Limitations

The main limitation of this study is its retrospective, observational nature. Only patients who underwent surgery provide any information, since patients removed from the waiting list without surgery have no chance of contributing their waiting times to this analysis [31].

Another potential source of limitation of the study is the elevated number of missing values for educational level, although the sensitivity analyses show that the risk of bias due to the presence of missing values and possibly due to the record linkage process is very low.

Lastly, our definition of waiting time does not take into account the time between the general practitioner referral and the consultation.

## Conclusion

This study provides evidence on the effect of socioeconomic status on waiting times for elective surgery by using individual records and administrative databases. Inverse trends were observed between educational level and waiting times for all procedures except for CABG. The results of the study suggest the need for implementation of key policies aimed at promoting regional information initiatives to guarantee wider access to those with a low socio-economic status by increasing their knowledge of available services and their ability to act.

## Endnote

^a^Conventional Cox proportional hazard estimates are available from the authors upon request.

## Competing interests

The authors declare no competing interests.

## Authors’ contributions

AP conceived of and designed the study. GDL participated in the design of the study, performed the statistical analysis, interpreted the findings and drafted the manuscript. TL created the study analytic dataset and performed the statistical analysis. GC conceived of the study. All authors critically read, revised and approved the final manuscript.

## Pre-publication history

The pre-publication history for this paper can be accessed here:

http://www.biomedcentral.com/1472-6963/12/268/prepub
